# Extracellular matrix from decellularized porcine organs as scaffolds for insulin-secreting cells and pancreatic islets

**DOI:** 10.3389/fendo.2025.1722536

**Published:** 2026-01-19

**Authors:** Vignesh Dhandapani, Pakindame Boabekoa, Patrick Vermette

**Affiliations:** 1 Laboratoire de bio-ingénierie et de biophysique de l’Université de Sherbrooke, Department of Chemical and Biotechnological Engineering, Université de Sherbrooke, Sherbrooke, QC, Canada; 2 Centre de recherche du Centre hospitalier universitaire de Sherbrooke (CHUS), Faculté de médecine et des sciences de la santé, Sherbrooke, QC, Canada

**Keywords:** decellularized porcine organs, endothelial cells, extracellular matrix (ECM), insulin secretion, pancreatic mouse islets, tissue engineering

## Abstract

Extracellular Matrix (ECM) from different organs has been used to cultivate several cell types. ECM produced by organ decellularization contains collagens, fibronectin, glycosaminoglycans (GAGs), laminins and other components essential in providing structural support and biochemical cues for cells to attach, function, and proliferate. The organ from which ECM is extracted and produced is hypothesized to play a vital role in cell responses upon recellularization. To investigate this hypothesis, five porcine organs (bladders, kidneys, livers, lungs, and pancreas) were decellularized by a detergent-based method or by detergent-free procedures. Insulin-secreting rat pancreatic β-like cells (INS-1) were first used to screen, over 7 days, the effect of the ECM produced by the tested decellularization techniques from the five selected organs, revealing SDS treatment did not result in cell responsive ECMs for all the tested organs. Detergent-free-derived ECMs, on the other hand, allow cell attachment except for the pancreatic ECM. The biocompatibility of the ECMs made from detergent-free methods was subsequently validated using cell proliferation and cell metabolism assays, immunostaining for insulin and actin expression, as well as glucose-stimulated insulin secretion (GSIS). INS-1 cells proliferated on certain detergent-free ECMs and secreted insulin following 7 days of culture. Further, primary pancreatic mouse islets were isolated and cultivated 48 hours on detergent-free decellularized bladder pieces and histological analysis showed intact islets embedded within the bladder ECM. GSIS revealed functional islets following 48 hours on detergent-free-derived bladder ECM. Islets cultivated on detergent-free-derived bladder ECM expressed insulin, with endothelial cells (i.e., CD31-positive cells) localized at the islet-ECM interface.

## 1 Introduction

Tissue engineering methods often involve the use of scaffolds and biological molecules to support cells to fabricate tissues or organs *in vitro* and/or *in vivo*, yielding a product with a range of biological properties to meet the targeted application requirements. Scaffolds composed of or containing ExtraCellular Matrix (ECM) are of great interest owing to the ECM unique (ultra)structure and composition, making ECM the gold standard for scaffolds. The ECM is a heterogenous fibrous network of glycoproteins structurally and functionally coordinated, providing mechanical stability and acting as a physical scaffold, as well as offering biochemical cues essential for tissue morphogenesis and homeostasis (1). Decellularization of tissues and organs to produce ECMs has gained interest in the last decade, although soft tissues were decellularized as early as in the 1970s.

Briefly, organ decellularization refers to the process of removing cellular components from tissues or organs leaving a network of proteinaceous ECM. Although different decellularization techniques i.e., physical, chemical and/or enzymatic, have been applied to decellularize tissues and organs with different efficiencies, often a combination of those techniques has been successful in removing cellular components and preserving ECM (ultra)structure. The impact of the applied decellularization method on the resulting matrix (ultra)structure is described elsewhere (2). Basement membrane proteins (collagen IV and laminins), fibronectins and glycosaminoglycans have been evolutionarily conserved, thereby eliciting similar cellular responses among xenogenic and allogenic scaffolds when implanted *in vivo* or seeded with cells *in vitro* (3, 4). On the other hand, composition as well as structural and mechanical properties of the ECM vary among different organs because of the difference in the structural and functional molecules secreted by the organ resident cells (5, 6).

A variety of products derived from ECMs are emerging. Tissue papers fabricated from decellularized porcine and bovine organs aided in the proliferation of human mesenchymal stem cells (7). Recently, 3D bioprinting with ECM embedded in scaffolds shows potential because of the ability to customize scaffolds architecture with the ECM (8). Hydrogels derived from ECM were able to aid in bone and cartilage engineering (9), fibroblast proliferation (10) and human corneal stromal cell attachment (2). Several products have been derived from decellularized animal and human organs and are already commercialized. For example, Oasis^®^ (porcine small intestine submucosa, Cook Biotech Inc. Indiana, USA), MatriStem^®^ (urinary bladder mucosa, ACell, Columbia, USA), and Restore™ (porcine small intestine, DePuy Othopedics Inc., Indiana, USA) (11) are commercially available.

The bladder has been one of the most used tissue to extract and produce ECM to fabricate scaffolds and to cultivate cells, as exemplified by the bladder ECM used to support the proliferation of urothelial cells (12), human bone marrow stem cells, human muscle progenitor cells and fibroblasts (13). Probably, one of the reasons is the good yield following decellularization. The use of decellularized urinary bladder-derived matrices to cultivate different cell types prompted us to carry out a study aiming to compare a single cell type or tissue response over ECM produced from different decellularized organs. This would allow investigating the potential, or limitations, to use other organs as ECM sources. Although, few studies have reported comparison of cell responses on different decellularized organs, most of those studies have been conducted on modified ECMs such as ECM-derived or ECM-containing hydrogels, tissue papers and 3D printed scaffolds. In the present study, it was decided to compare cell responses keeping the ECM as intact as possible to preserve as much as possible the ECM composition and (ultra)structure, making our study challenging experimentally, but unique.

An increase in the number of type 1 diabetes cases globally and the associated complications such as ischemia, loss of islet viability, hypoglycemia and harmful effects of long-term immunosuppression prompted the need for an alternative approach. Tissue engineering approaches using decellularized organs to tackle the issue can be an option. An earlier study showed that the porcine bladder ECM obtained by detergent-free decellularization was able to aid in the attachment, proliferation and functionality of insulin-secreting pancreatic cells (INS-1) (14). The objective of this study is an extension of a previous one comparing INS-1 cell responses on ECMs extracted and produced from five decellularized porcine organs (bladders, kidneys, lungs, livers, and pancreas). The five organs were used considering the availability of the organs and the available literature supporting the potential of these organ ECMs. This first part of the study allowed us to screen and select the ECMs showing the most promising outcomes to then investigate the effects on primary mouse islets. The organs were decellularized by four methods of which, three methods involved detergent-free decellularization and one, the use of 0.5% sodium dodecyl sulfate (SDS) as the detergent. INS-1 cells were seeded on the ECM and grown for 7 days. Cell viability was visualized by a metabolism assay (MTT) and cell proliferation investigated using the CyQUANT test. Functionality was characterized by glucose-stimulated insulin secretion (GSIS) and immunostaining for insulin. Mouse primary pancreatic islets were then isolated and seeded on the detergent-free decellularized bladder ECM and functionality characterized by glucose-stimulated insulin secretion (GSIS) and immunostaining for insulin and endothelial cells.

## 2 Materials and methods

### 2.1 Organs decellularization

Porcine organs (bladders, kidneys, livers, lungs, and pancreas) from three different porcine donors (N = 3) were freshly obtained from the slaughterhouse (Abattoir Régional de Coaticook, Coaticook, Québec, Canada) within 24 hours of animal sacrifice. The porcine organs were procured from a licensed slaughterhouse and since the animal sacrifice was performed under strict regulated conditions, the procedure needed no approval from the ethics committee of the Université de Sherbrooke. They were placed on ice until further processing. Briefly, the bladder, being muscular, was delaminated and the resulting tissue was cut into pieces of approx. 5 mm x 5 mm. The other organs were diced into smaller pieces and fed into a meat grinder (Heavy-duty Electric Meat Grinder, Model #8 ¾ HP Motor, Weston). The disrupted organ was collected and subjected to detergent-based or detergent-free treatments (14, 15).

Briefly, for the detergent-based method, 0.5% Sodium Dodecyl Sulfate (SDS) (161-032, Biorad) was used to decellularize the organs for 30 ± 1 hours at 200 rpm in a shaker (Innova^®^ 44/44R, New Brunswick™). The decellularized samples were washed again in distilled water for 48 hours (4 changes of water) and the final water wash contained 1% penicillin/streptomycin (15140122, Life Technologies). The decellularized organs were filtered and stored at -20˚C until further use.

The first applied detergent-free method involved two cycles of freeze-thaw and treatment with 2M sodium chloride. The second detergent-free method was supplemented with a 0.5% ethylene diamine tetra acetic acid (EDTA) treatment while the third was complemented by a pH adjustment to reach a pH of 6.6. Finally, all the samples were washed and stored at -20˚C until further use.

### 2.2 Cell culture setup and INS-1 cell culture

2% agarose (A0169, Sigma-Aldrich) solutions were prepared in either

- INS-1 cell culture medium composed of RPMI-1640 (31800-022, Life Technologies) supplemented with 10 mM HEPES (BP310, Thermo Fisher Scientific), 10% foetal bovine serum (FBS) (12483020, Life Technologies), 1 mM sodium pyruvate (11360-070, Gibco), 50 µM β- mercaptoethanol (M7522, Sigma-Aldrich), and a 1% penicillin/streptomycin mixture, or
- Islet culture medium made with 1:1 of DMEM (D5523, Sigma-Aldrich, low glucose):RPMI (R1383, Sigma-Aldrich) supplemented with 5% FBS, 15 mM HEPES, 10 mM nicotinamide and a 1% penicillin/streptomycin mixture.

The agarose gels made from INS-1 cell culture medium and islet culture medium were used to culture INS-1 cells and islets, respectively. Firstly, layers of agarose resulting from 300µL of the agarose solution were made at the bottom of the wells of 24-well cell culture plates. Porcine organ ECM pieces were transferred to each well covering ca. 90% of the total surface area. A second layer of agarose was coated on the sides of the ECM (not covering the ECM pieces) to stick it to the first agarose layer, as described in a previous study (14). Wells with the ECM were soaked in 1X PBS for 90 minutes and simultaneously asepticized under the laminar hood UV-light. The whole setup was soaked in sterile RPMI-1640 or DMEM medium for the culture of INS-1 cells or islets, respectively for 48 hours at 37˚C and 5% CO_2_.

Stock INS-1 cells (C0018007, AddexBio) were grown in INS-1 cell culture medium in T75 flasks at 37˚C and 5% CO_2_. Upon reaching confluence, cells were trypsinized (25200072, Life Technologies), counted and 25, 000 cells per well were added. INS-1 cells passaged for 16–25 times were utilized for the experiments. Media were changed every 48 hours and cells were grown for 7 days.

### 2.3 Cell proliferation quantification

INS-1 cells grown on ECMs for 4 hours (the time needed for INS-1 cells to attach to the ECM) and 7 days, as well as negative controls (i.e., wells with ECMs but with no cells), were quantified using the CyQUANT™ NF Cell Proliferation Assay Kit (C35006, Thermo Fisher Scientific). Briefly, the media from different wells were conserved in separate vials. Cells from each well were isolated by trypsinization (6–7 minutes) and vigorous repeated pipetting. They were transferred to vials containing media and centrifuged at 1200 rpm for 5 minutes. The supernatant media were discarded, the pellets rinsed in 1X Hanks Buffered Salt Solution (HBSS), centrifuged again and the supernatant discarded. Working concentration of CyQUANT™ NF reagent was prepared according to the protocol provided by the manufacturer, added to the cell pellet, and transferred to a 96-well microplate. The plate was incubated for 1 hour at 37˚C and the fluorescence was read at 480 nm (Synergy HT Microplate Reader, Biotek).

### 2.4 Visual observation of viable cells on ECM pieces

A solution of 0.5 mg/mL thiazolyl blue tetrazolium bromide (M5655, Sigma-Aldrich) was prepared in 1X HBSS. The medium from each well was discarded and 1 mL of the prepared 3-(4, 5-dimehtylthiazol-2-yl)-2, 5-diphenyltetrazolium bromide (MTT) solution was added in each well. The plate was incubated at 37˚C for 1 hour under 5% CO_2_ humidified conditions. Following incubation, the MTT solution was discarded and 1X PBS was added to the wells. A stereomicroscope (Leica MZFLIII, Germany) was used to visualize the cells and capture the images.

### 2.5 Islet isolation and culture

Adult mice (CD-1^®^ IGS, Charles River, Boston, MA, USA) were euthanized under a CO_2_ atmosphere according to the approved protocol (# 367-14) from the Université de Sherbrooke. The pancreas was injected with the dissociation solution consisting of 2.5 mg/mL collagenase (C9263, Sigma-Aldrich) through the pancreatic duct. The dissociation solution contained L-15 medium (L-4386, Sigma-Aldrich) supplemented with 10 mM nicotinamide, 15 mM HEPES, 2% FBS, 0.35 mg/mL sodium bicarbonate, 1 mg/mL glucose and a 1% penicillin/streptomycin mixture. The pancreas was excised off the mouse and transferred on ice to the laboratory in a Falcon tube containing the dissociation solution. The tube was placed at 37˚C for 18 ± 2 minutes with manual shaking every 2 minutes. Ice-cold neutralization medium composed of HBSS (H1387, Sigma-Aldrich), supplemented with 5% FBS, 10 mM nicotinamide and 0.35 mg/mL sodium bicarbonate, was added to stop the collagenase digestion. The whole digest was poured into bacteriological Petri dishes and islets were handpicked under a stereomicroscope (Carl Zeiss, SteREO Lumar. V12, Germany) and transferred to vials containing the dissociation solution with no collagenase. Islets were centrifuged at 1200 rpm for 1 minute and resuspended in islet culture medium. Approximately 22 islets/well were seeded onto the soaked bladder ECM pieces or on Tissue Culture Polystyrene (TCPS, 24-well plates). Islets were cultivated for 48 hours considering the goal was to investigate the effect of seeding on insulin secretion. We have established that 48 hours was the minimal duration for islets to adapt to the condition i.e., interact with the material, and secrete insulin (16). The islets were isolated from the pancreas of three independent mice (N = 3).

### 2.6 Insulin and actin expression by immunofluorescence

ECM pieces seeded with cells and islets were fixed in 4% PFA for 48 hours. Samples were processed, dehydrated, embedded in wax, and sections of 4-µm thickness were cut. The dried sections were deparaffinized, hydrated and blocked with 2% bovine serum albumin. The primary antibodies to insulin (ab181547, Abcam) and β-actin (ab8226, Abcam) were added at the recommended dilution and left undisturbed overnight at 4˚C to the samples with INS-1 cells. The secondary antibodies anti-rabbit Alexa 488 (Invitrogen) and anti-mouse Alexa 647 (Invitrogen) were added and left for 1 hour at room temperature and were counterstained with 4, 6- diamidino-2-phenylindole, dichloride (DAPI, D1306, Invitrogen). For the islets, primary antibodies to insulin (ab181547, Abcam) were added and left undisturbed overnight at 4˚C. Secondary antibodies anti-rabbit Alexa 488 (Invitrogen) were added at the recommended dilution and, incubated at room temperature for 1 hour. Endothelial cells visualization was performed by staining for CD31 using a polyclonal goat IgG primary antibody (R&D systems, AF3628) and horse anti-goat Dylight™ 488 (Vector laboratories, DI-3088-1.5) as secondary antibody. Two fluorescence images from four different samples in each case were visualized using an Olympus IX83 inverted confocal microscope (Olympus life sciences, PA, USA) at 20x and images were captured using the software Olympus FV-31S-SW.

### 2.7 Histological characterization

ECM pieces seeded with islets cultivated for 48 hours were fixed in 4% PFA for 48 hours. Samples were processed, dehydrated, embedded in wax, and sections of 4-µm thickness were cut. Sections were dried, deparaffinized and hydrated. Sections were characterized using Hematoxylin and Eosin (H&E) staining to confirm the presence of islets embedded within the ECM. Images were acquired in visible mode at a 20x magnification using a NanoZoomer 2.0-RS slide scanner (Hamamatsu Photonics K.K, Bridgewater, NJ, USA) for digital pathology.

### 2.8 Functionality investigation by glucose-stimulated insulin secretion

The supernatant fluids from 7-day INS-1 cell cultures and 48-hour mouse pancreatic islet cultures were discarded after centrifuging the media. 1X PBS was used to wash the pellet, centrifuged, and the supernatant fluid discarded. The cell pellet with the entire well content was washed twice (30 minutes and 1 hour of incubation) in a low-glucose (2.8 mM) solution containing Krebs-Ringer buffer (KRBH) supplemented with 115 mM NaCl, 5mM KCl, 25 mM HEPES, 24 mM NaHCO_3_, 5mM CaCl_2_, 1mM MgCl_2_ and 0.1% bovine serum albumin to remove the residual insulin already present in the system. Further, the whole well with the cells was incubated sequentially in low-glucose (2.8 mM, LG1), high glucose (28 mM, HG), high glucose ((28 mM) + 50µM 3-isobutyl-1-methylxanthine (IBMX, I5879, Sigma-Aldrich), HG+IBMX) and low-glucose (2.8 mM, LG2) for 1 hour at 37˚C. KRBH buffer was used to prepare the glucose solutions. Post-incubation in each step, the buffer was collected, centrifuged and the supernatant fluid (containing the insulin) stored at -20˚C. Rinsing of the wells and cell pellets with 1X PBS following centrifugation was done in between each step with the rinsing solution discarded every time to remove residual insulin from the previous step. Insulin-containing solutions were stored at -20˚C until running ELISA. Rat High Range Insulin ELISA (80-INSRTH-E01, Alpco Diagnostics) was used to estimate the insulin concentration from INS-1-seeded samples and Mouse High Range Insulin ELISA (80-INSMSH-E01) for islet-seeded samples. The secreted insulin was normalized to write 100,000 cells and per islet, respectively, and the stimulation index was calculated for each condition by dividing the insulin concentration measured under high-glucose stimulation to that under low-glucose stimulation.

### 2.9 Statistical analysis

One or two-way analysis of variance (ANOVA) or unpaired t-test was performed using GraphPad Prism with Šidáks multiple comparison test. Microsoft Office 365 Excel was used to plot the graphs. A p value < 0.05 was considered statistically significant. The bars in the graph represent mean of three experiments ± standard error.

## 3 Results and discussion

### 3.1 Proliferation of INS-1 cells on ECMs

Characterization of ECMs derived from the different organs following detergent-free and detergent-based decellularization is described elsewhere (15). ECMs were characterized for the removal of cellular components and dsDNA, as well as protein composition.

**Figure 1:**
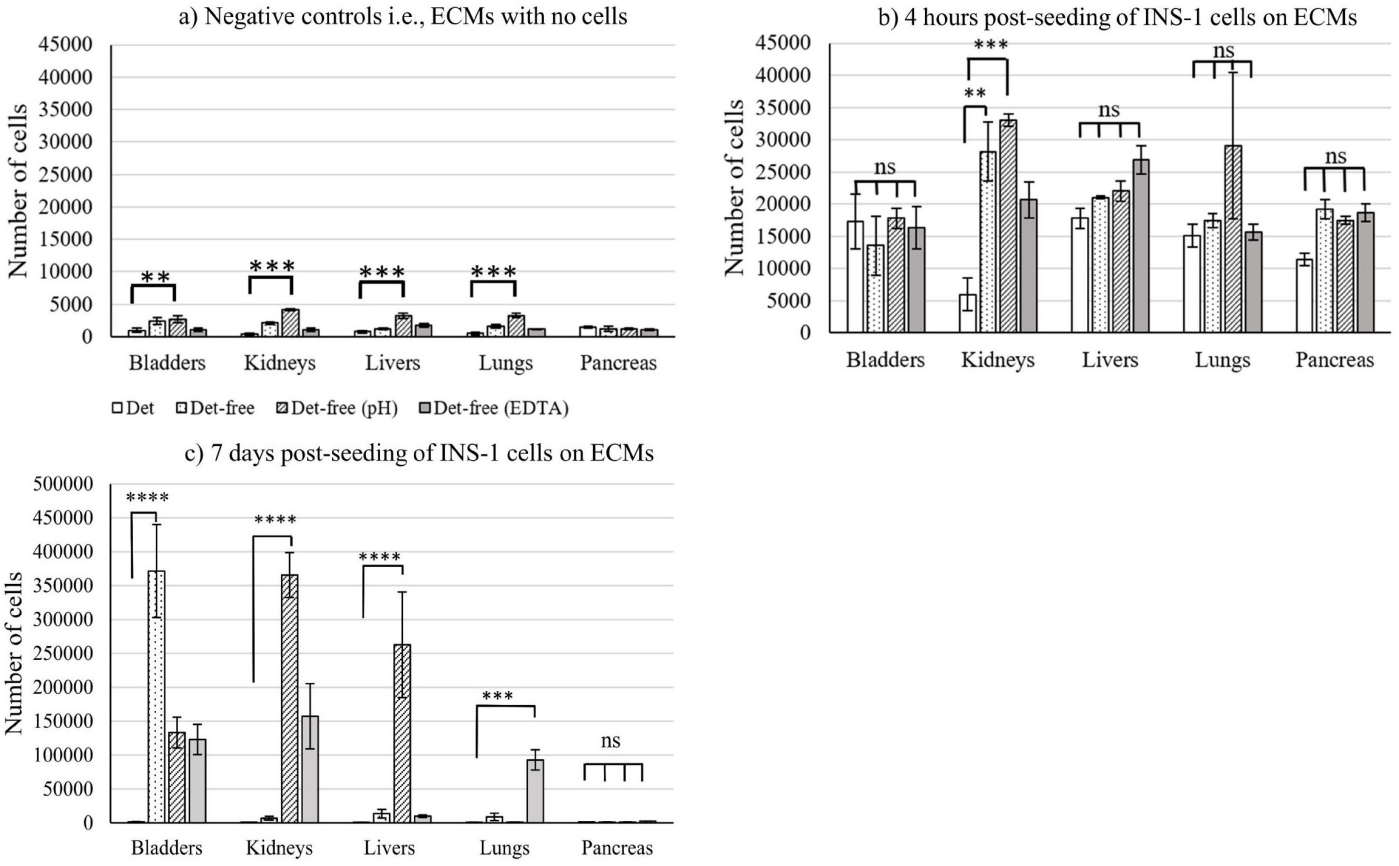
Number of INS-1 cells derived from the CyQUANT assay for **(a)** ECMs with no cells as well as **(b)** 4 hours, and **(c)** 7 days post-seeding of INS-1 cells on ECMs. Statistical analysis was done comparing detergent-based decellularization to the detergent-free ones. The data for the INS-1 cell number seeded on detergent-based and detergent-free alone decellularized bladder has been reported in an earlier study and has been used here to compare with other detergent-free decellularized bladders and other organs (14). The data reported here are gathered from three different porcine donors (N = 3). Tukey’s two-way ANOVA was used to analyze significance. **** corresponds to p<0.0001, *** represents p<0.001, ** represents p<0.01, * represents p<0.05 and ns corresponds to non-significant. Bars represent average ± S.E. Initial cell seeding density is write 25,000 cells per well.

Cell proliferation following 7 days of culture indicated that INS-1 cells proliferation was higher on bladders decellularized by the detergent-free method, as compared to cells on the other organs (**Figure 1**). ECMs produced from bare detergent-free decellularization (i.e., with no additional treatment) did not result in cell proliferation in all cases, except for bladders. But INS-1 cells proliferated on detergent-free decellularized (alone, with pH and ethylene diamine tetraacetic acid (EDTA) treatments) bladders, (pH- and EDTA-treated ECM) kidneys, (pH-treated) livers, and (EDTA-treated) lungs. Cell proliferation was not observed on decellularized pancreas, in all conditions. This could be due to the pancreas being necrotic on isolation due to the presence of exocrine enzymes, potentially digesting the pancreas upon isolation (15). This points out to the absolute need to use freshly harvested (i.e., less than one hour) pancreas for decellularization. Even if the organs and the pancreas were obtained from the slaughterhouse less than 24 hours following animal sacrifice, this was not good enough for pancreas. Importance of pancreas preservation prior to its *in vivo* isolation and the need for qualified surgeons for transplants are reported elsewhere (17). Cell proliferation on ECMs extracted using detergent-free processes (EDTA and/or pH treatments) for bladders, livers, lungs, and kidneys was significant as compared to detergent-based ECMs, where the cells did not proliferate. The negative controls without cells in **Figure 1a** indicated that the CyQUANT signals did not arise from the ECM itself. The number of cells after 4-hour seeding indicated viable cells on the different ECMs compared to the negative controls, as shown in **Figure 1b**. In summary, cell proliferation analysis indicates that INS-1 cells proliferated on at least one of the ECMs derived from detergent-free decellularization for bladders, lungs, livers, and kidneys but not for the pancreas ECM.

Basement membrane proteins such as collagens, laminins, heparan sulphate and fibronectin are essential for β-cell survival and proliferation (18). Of all the collagens, collagen IV is key for INS-1 cells and MIN-6 cells proliferation (19, 20). Biomimetic materials incorporating decellularized urinary bladders have been used to cultivate fibroblasts (10), adipose-derived stem cells (21), and β-like rat cells (14). Acellular kidneys were able to support the proliferation of embryonic stem cells (22), epithelial and endothelial cells (23) and primary renal cells (24). Decellularized livers have been repopulated with primary hepatocytes (25), mesenchymal stem cells (26), and endothelial cells (27). Decellularized lungs have been recellularized with lung fibroblasts, mesenchymal stem cells, and small airway epithelial cells (28). Some studies describe the recellularization of pancreas with INS-1 cells, primary islets (16) and mesenchymal stem cells (29), but all of those studies indicated excision of fresh pancreas with decellularization initiated within 1 hour of excision. Again, our results validated the necessity of rapidly using the pancreas to perform decellularization. This was not the case for the other four tested organs (bladders, kidneys, livers, and lungs), as histological analysis revealed no obvious signs of damage, as it was observed for the pancreas (15).

**Figure 2:**
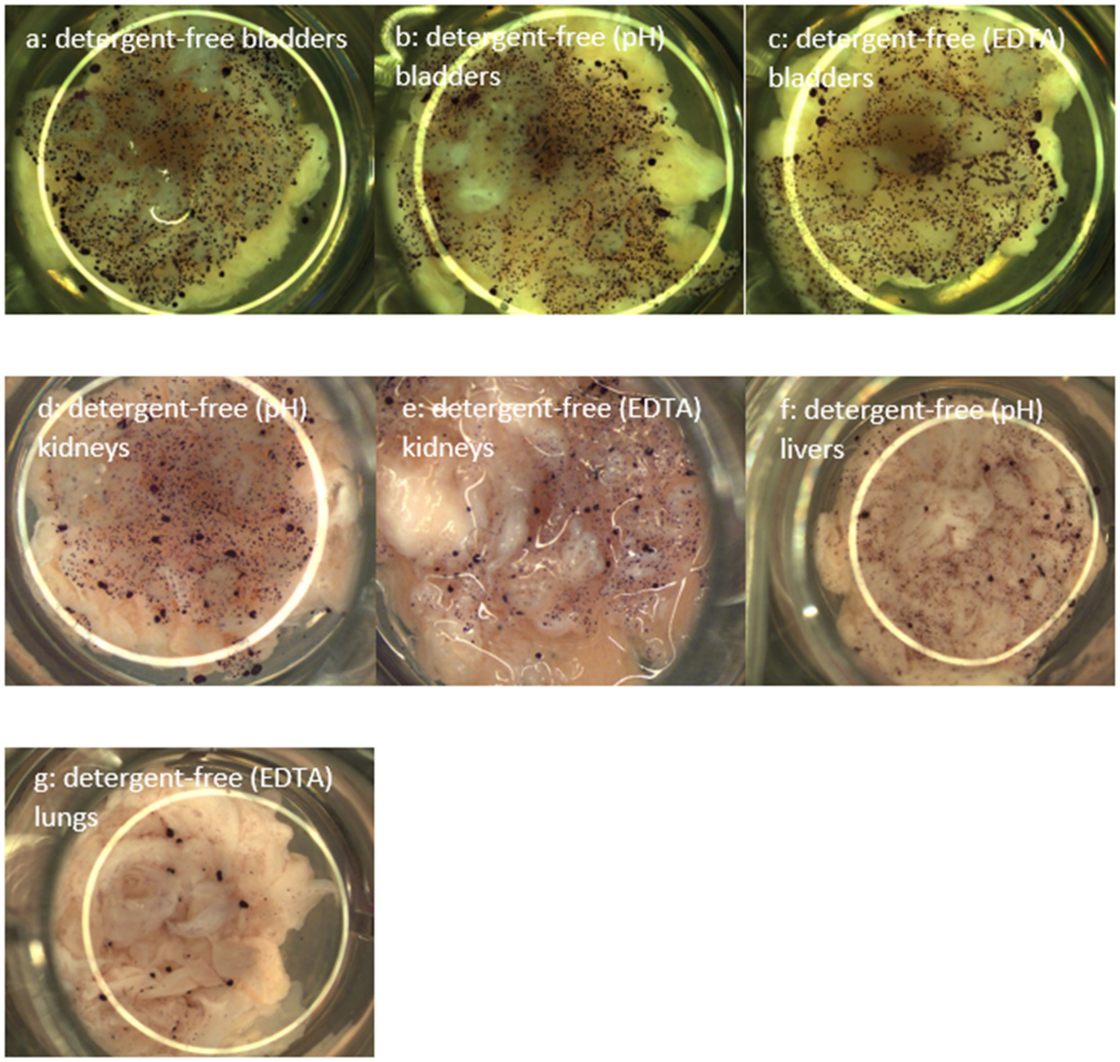
MTT staining allowing visualizing viable INS-1 cells on decellularized **(a)** detergent-free bladders, **(b)** detergent-free (pH) bladders, **(c)** detergent-free (EDTA) bladders, **(d)** detergent-free (pH) kidneys, **(e)** detergent-free (EDTA) kidneys, **(f)** detergent-free (pH) livers, and **(g)** detergent-free (EDTA) lungs following 7 days of cell culture. Representative images are shown in the figure; the data were validated on three different porcine donors (N = 3).

The metabolic activity of INS-1 cells, visualized by MTT staining following 7 days of culture, supported CyQUANT cell proliferation results. Purple-colored crystals were visualized in all conditions where cells have proliferated, pinpointing metabolically active cells, as shown in **Figure 2**. ECMs where cells did not proliferate and negative controls were negative in terms of metabolic activity, as no purple-colored crystal was observed. MTT assay was successfully used to observe cell proliferation of human endometrial mesenchymal cells seeded on decellularized mouse liver (30). The MTT assay was applied here to visually confirm cell viability on the non-transparent samples and to supplement the CyQUANT proliferation analysis.

### 3.2 Functionality of INS-1 cells on ECMs

**Figure 3:**
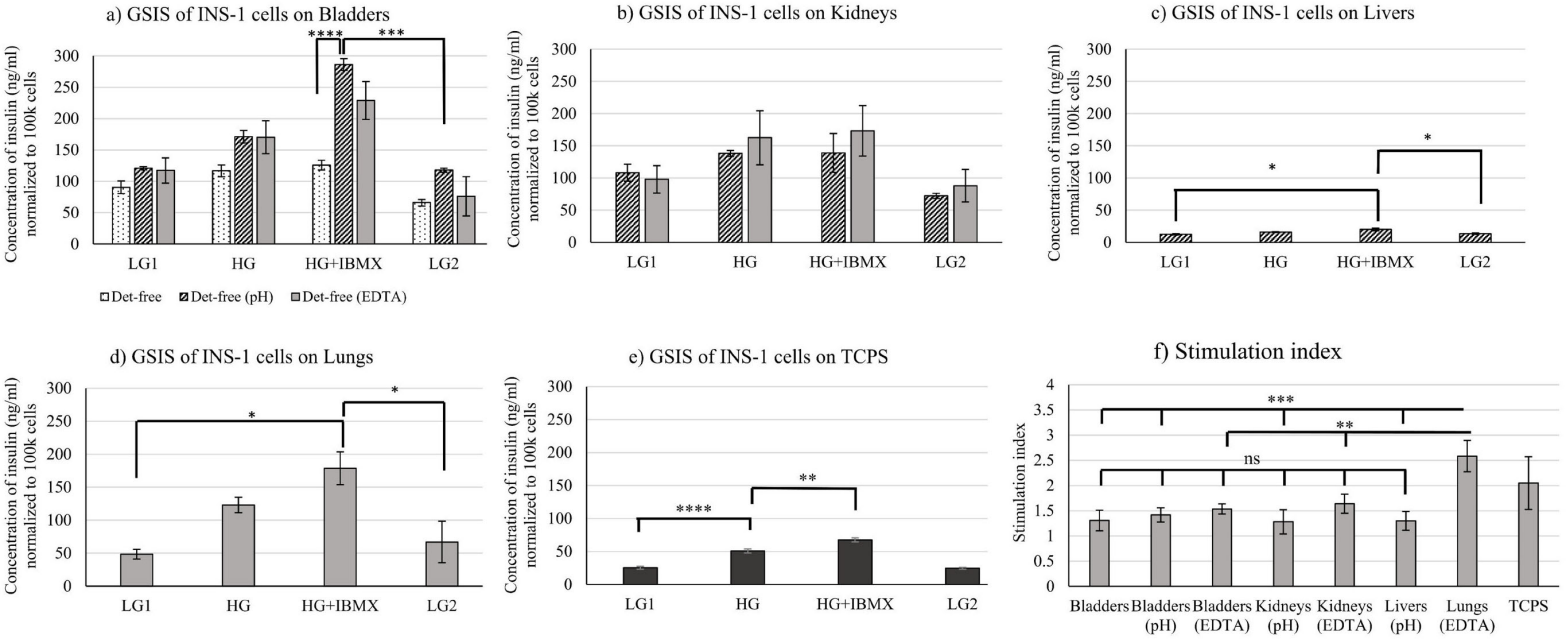
Functionality assessed by glucose-stimulated insulin secretion (GSIS) of INS-1 cells cultured for 7 days on decellularized **(a)** bladders, **(b)** kidneys, **(c)** livers, **(d)** lungs, and **(e)** TCPS. **(f)** Stimulation index in each case (Stimulation index S.I. = concentration of insulin at HG/concentration of insulin at LG1). Glucose-stimulated insulin secretion data for INS-1 cells seeded on detergent-free alone decellularized bladders has been reported in an earlier study and has been used here to compare with other detergent-free decellularized bladders and other organs (14). Data reported here are gathered from three different porcine donors (N = 3). Ordinary one-way ANOVA with Šidáks multiple comparisons tests was used to determine significance. p<0.05 was significant and indicated by *, p<0.01 indicated by **, p<0.001 indicated by *** and p<0.0001 indicated by ****. TCPS means Tissue Culture Polystyrene. Bars represent average ± S.E.

ECMs on which cells have proliferated also supported INS-1 cell functionality, as revealed by measuring insulin secretion after glucose stimulation (**Figure 3**).

Comparing insulin secretion of INS-1 cells on Tissue Culture Polystyrene (TCPS), insulin secretion was higher for INS-1 cells on ECMs in all conditions, except for the detergent-free (EDTA) livers (**Figures 3a–e**). Cells seeded on decellularized detergent-free bladders (bare, pH and EDTA), kidneys (pH and EDTA), livers (pH), and lungs (EDTA) were functional, as shown in **Figures 3a–d**. Since cells were metabolically inactive on the rest of the ECMs, confirmed by the MTT test, GSIS was not performed for the cells seeded on those ECMs. A previous study described the conservation of collagen-4 and laminins in detergent-free decellularized bladders (bare, pH and EDTA) (15). Basement membrane proteins such as type-4 collagen and laminins have been described to play a key role in insulin secretion from human islets (31). This could explain the reason for insulin secretion by INS-1 cells seeded on detergent-free decellularized bladders, as observed in **Figure 3a**. Collagen-4 was not conserved in the detergent-free decellularized (bare, pH and EDTA) livers, whereas laminins were conserved in the detergent-free decellularized (pH) livers in an earlier study (15). Reduced insulin secretion with decellularized liver in comparison to the other organs was observed in **Figure 3c**, corresponding to the proteomic results from our recent study.

The stimulation index *i.e*., the ratio of insulin concentration at high-glucose stimulation to that at low-glucose, was higher for cells on TCPS, as shown in **Figure 3e**, compared to all conditions except for the detergent-free (EDTA)-treated lungs. The stimulation index of INS-1 cells seeded on decellularized lungs (EDTA) was significantly higher than those of the other conditions, as shown in **Figure 3f**. The stimulation index of INS-1 cells on TCPS, as shown in **Figure 3f**, was comparable to that of a culture on fibronectin-coated plates (32). Stimulation indexes of cells in all conditions, except that for the detergent-free (EDTA) lungs, was comparable to that of INS-1 cells grown in fibrin for 48 hours (33). In a previous study, the stimulation index observed in 3D hydrogels was lower, as compared to that with 2D cultures (34). However, it is risky to compare the two systems (TCPS and 3D cultures), as 3D culture systems can create a diffusional barrier and/or can result in trapped insulin content (16, 35). The cell-matrix interactions influenced the survival and insulin secretion of β-cells by activation of NF-κB signaling (36–38). Collagen IV, laminins, fibronectins and other ECM proteins were conserved in detergent-free decellularized organs, as characterized by mass spectrometry (15). Laminins in the basement membrane are key proteins responsible for insulin gene expression and β-cell proliferation (39). Laminins were conserved in the detergent-free decellularized ECMs and could have supported insulin expression and thereby functionality (15).

Immunostaining for insulin in INS-1 cells has revealed green-coloured droplets of insulin, as shown in **Supplementary Figure 1**. This supports that cells were functional on the screened ECMs. The staining for β-actin revealed the cytoskeleton of the cells, as shown in **Supplementary Figure 1**. Focal cell adhesion points i.e., areas at which the cell interacts with the ECM, were observed as extensions of actin filaments and shown in **Supplementary Figure 1**. This is in accordance with another study reporting INS-1 cells on decellularized bladders (14). Immunostaining indicated higher insulin fluorescence (green-droplets) on detergent-free decellularized bladder ECMs (bare, EDTA, pH) as compared to the insulin fluorescence on other organ ECMs (**Supplementary Figure 1**). These results were coherent with the insulin secretion results, as shown in **Figures 3a–d**. Higher insulin secretion was observed for the INS-1 cells cultivated on detergent-free decellularized bladders compared to other organ ECMs at low and high concentrations of glucose. Since cells were metabolically inactive and did not proliferate on the rest of the ECMs, confirmed by the MTT test and CyQUANT assay, immunostaining was not performed for the cells seeded on those ECMs.

### 3.3 Functionality of pancreatic mouse islets seeded on detergent-free-produced bladders ECM

**Figure 4:**
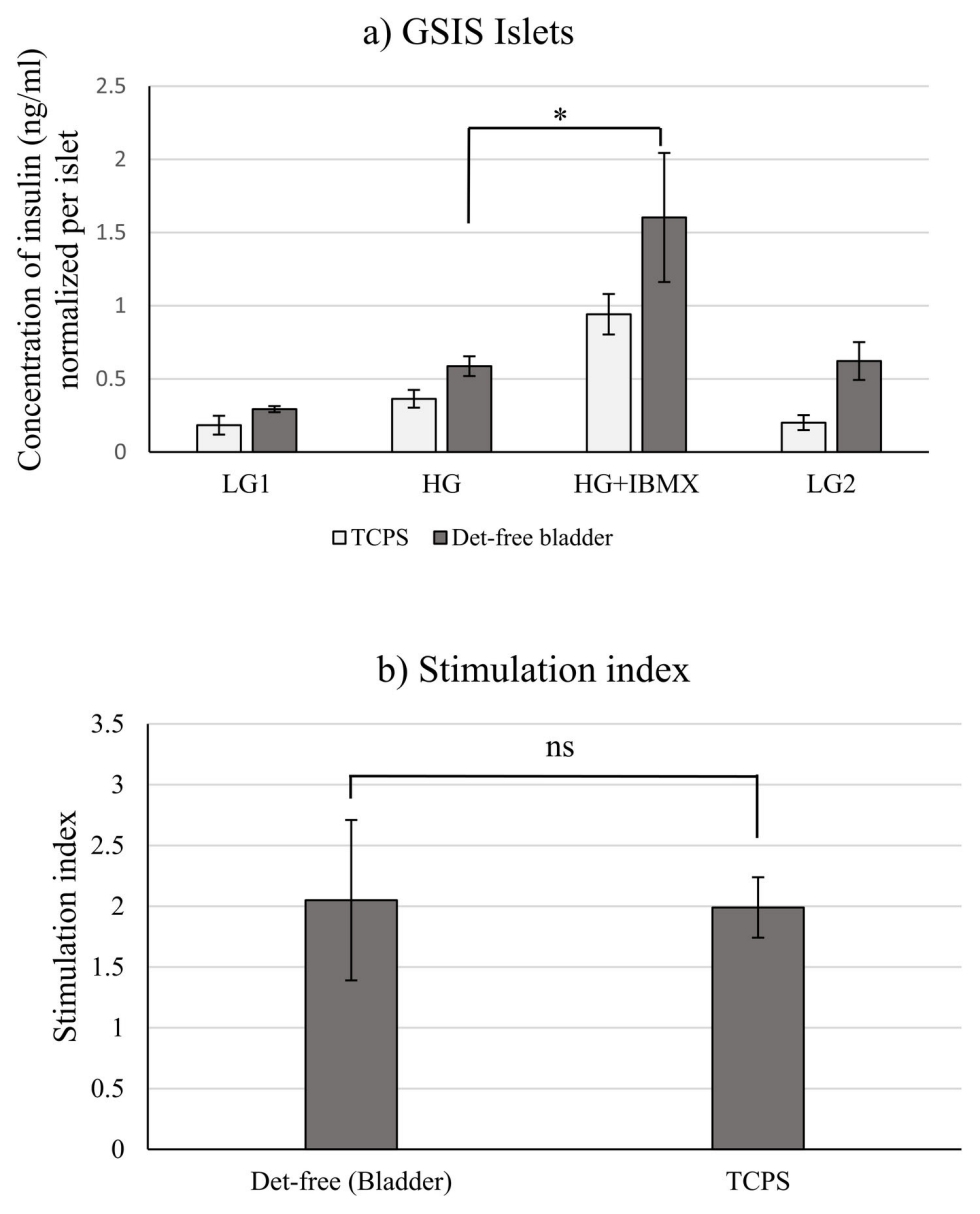
**(a)** Glucose-stimulated insulin secretion (GSIS) from islets cultivated 48 hours on TCPS and bladders ECM obtained from the bare detergent-free decellularization method. **(b)** Stimulation index of islets. The data reported here are gathered from three different porcine donors (N = 3). Unpaired t-test was used to determine the significance. ns indicates no significance. Stimulation index = Concentration of secreted insulin at HG/Concentration of secreted insulin at LG1. TCPS refers to Tissue Culture Polystyrene. Bars in the graphs indicate **(a)** Average ± SE and **(b)** Average± SD. * indicates a p value <0.05.

Considering the yield following decellularization and cell proliferation (**Figure 1c**), as well as the functionality of INS-1 cells on the different organ matrices, detergent-free decellularized bladder ECM was selected to support mouse islets culture. Functionality of mouse islets measured by the GSIS assay is shown in **Figures 4a, b**. As with INS-1 cells, islets were functional on the bladder ECM produced using the detergent-free method. The insulin secretion trend was as expected for the glucose concentrations used for stimulation. Previously, porcine (40) and rat (41) islets were used to recellularize decellularized pancreas. Although islets were seeded in the decellularized pancreas, the functionality was not reported. Later, a study reported a similar trend for islets seeded into decellularized mouse pancreas (16). A glucose concentration of 28mM was used here as the high-glucose concentration in the GSIS, because maximum insulin secretion from islets isolated from rodents was reported at glucose concentrations above 28mM (42, 43). Islets seeded on ECM obtained from decellularized bladders secreted more insulin, as compared to those on TCPS. The stimulation index was slightly higher for the TCPS, as compared to that of cells on bladder ECM.

**Figure 5:**
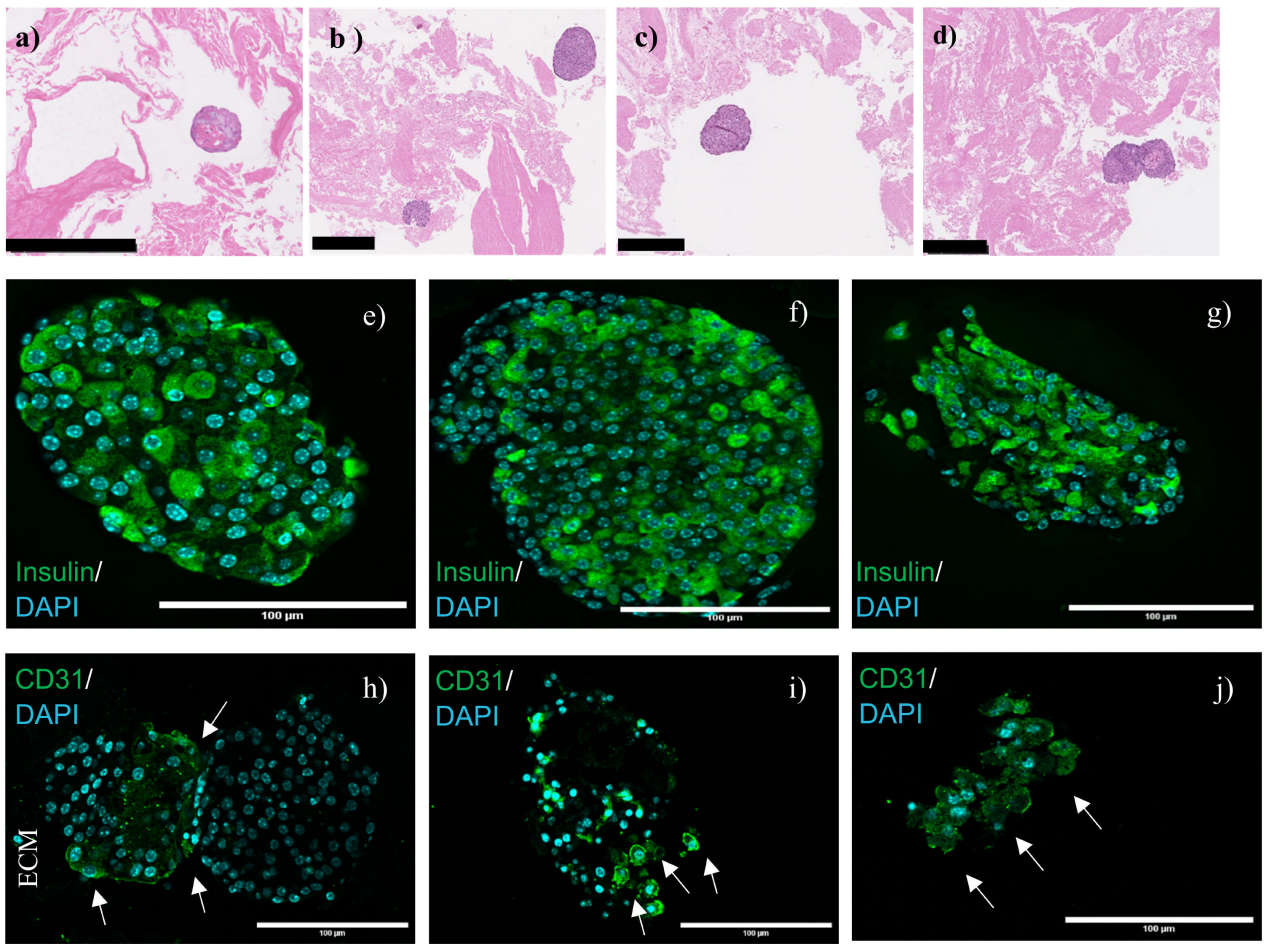
Islets cultivated 48 hours on detergent-free decellularized bladders stained by Haematoxylin and Eosin (H&E) **(a–d)**, insulin expression **(e–g)** and endothelial cells (CD31-positive cells) **(h–j)**. White arrows indicate endothelial cells. Islets in panel h correspond to the islets in panel d to highlight the presence and position of the ECM. Black scale bars correspond to 250 μm and white scale bars to 100 μm. Representative images are shown in the figure; data were validated on three different porcine donors (N = 3).

The pancreatic duct is closely associated with the islets in adult rats and the ductal ECM contains laminins, collagen IV and fibronectin, all responsible for β-cell survival (44, 45). Mass spectrometric analysis of the bladder ECM revealed the conservation of the necessary proteins (laminins, collagen IV and fibronectin) and this could have supported islets functionality (15). The presence of islets on the seeded ECM following 48 hours of culture was confirmed using H&E staining, as shown in **Figures 5a–d**. Histological characterization revealed intact islets with intact nuclei in the cells. Islets functionality was visualized by the expression of insulin within the islets, as shown in **Figures 5e–g**. In a previous study, mouse pancreatic islets cultivated 48 hours in decellularized mouse pancreas secreted insulin (16). Staining for CD31 indicated the presence of endothelial cells, as pointed out in **Figures 5h–j**. Although not very prominent, endothelial cells were detected at the islet junction and near the ECM in **Figure 5h** and sparsely within the islets, shown by white arrows (**Figures 5i, j**). Islet capillaries consisting of endothelial cells are more extensive and crowded in rodents (46–48). They are responsible for the secretion of certain growth factors and endothelin-1, which in turn increases insulin secretion in mouse islets by triggering calcium influx into the β-cells (49, 50). Longer duration cultures would be essential to allow angiogenesis.

This study provides a preliminary assessment of various organ-derived ECMs and their biocompatibility following recellularization with insulin-secreting pancreatic cells and islets. Although data from three porcine donors are presented, larger sample sizes will be necessary to generate more robust data required to justify specific medical applications. Because the organs were sourced from a slaughterhouse, donor-to-donor variability and inherent differences in tissue characteristics could represent limiting factors. The functional assays were limited to a maximum data time point of 7 days and hence, long-term viability and immune response of cells and islets could not be fully supported. However, this study provides an initial screening of how different ECM sources influence pancreatic cells and islets. Future work to improve understanding of cellular responses to extracellular matrix could include quantitative proteomic analysis, mechanical analysis and *in vivo* implantation of ECM to better understand the clinical translational potential and biocompatibility of the matrix.

## 4 Conclusions

This study presents an overall comparison of the responses of a pancreatic β-like cell line (INS-1 cells) and mouse pancreatic islets towards ECMs obtained by decellularizing five porcine organs using three detergent-free methods and one detergent-based approach. The ECM derived from the bare detergent-free method supported cell proliferation and functionality. The conservation of laminins, collagen-IV, and fibronectin in ECMs produced using detergent-free methods could be a significant factor. The additional use of EDTA-chelation and isoelectric treatment by pH adjustment coupled to the bare detergent-free technique yielded cell-compatible ECMs (for bladders, livers, lungs, and kidneys).

Although numerous studies have reported methods for decellularizing tissues and organs, none have compared insulin secretion by pancreatic cells and islets cultured on different porcine organ-derived ECMs in their unsolubilized form. In fact, in this study the ECMs were not solubilized, enabling preservation of their native ultrastructure, which is an ECM feature known to influence cellular behavior. This work is unique in its comparative evaluation of cell responses towards organ-specific ECMs and may serve as a useful screening approach when considering scale-up and targeted applications.

This investigation is one of the very few comparing the response of a single cell type (β-like cells) on different organ ECMs. Screening of different organ ECMs for tissue engineering could reveal better suited ECMs developed and tuned for the targeted application such as insulin secretion, nerve regeneration, angiogenesis, etc. The investigation with primary pancreatic mouse islets showed that the ECM resulting from the detergent-free-treated bladders is suitable to obtain ECM supporting islet survival and functionality.

The present work also opens the door for the development of different organ ECMs for a multitude of products depending on the need and on the anatomical site of application. Pluripotent stem cells could benefit from a combination of different ECMs stemming from different organs to support their expansion and sequential differentiation. This could allow making functional biologically active scaffolds. Application-oriented products such as scaffolds, sheets, meshes and bioadhesives could be derived from ECMs extracted and formulated from different organs.

## Data Availability

The original contributions presented in the study are included in the article/supplementary material. Further inquiries can be directed to the corresponding author.
