# Supplementary material for: Extracellular matrix from decellularized porcine organs as scaffolds for insulin-secreting cells and pancreatic islets
Source: Front Endocrinol (Lausanne). 2026 Jan 19;16:1722536. doi: 10.3389/fendo.2025.1722536 (PMC12861875; doi:10.3389/fendo.2025.1722536)
Supplement: Supplementary file 1 [file Image1.pdf]

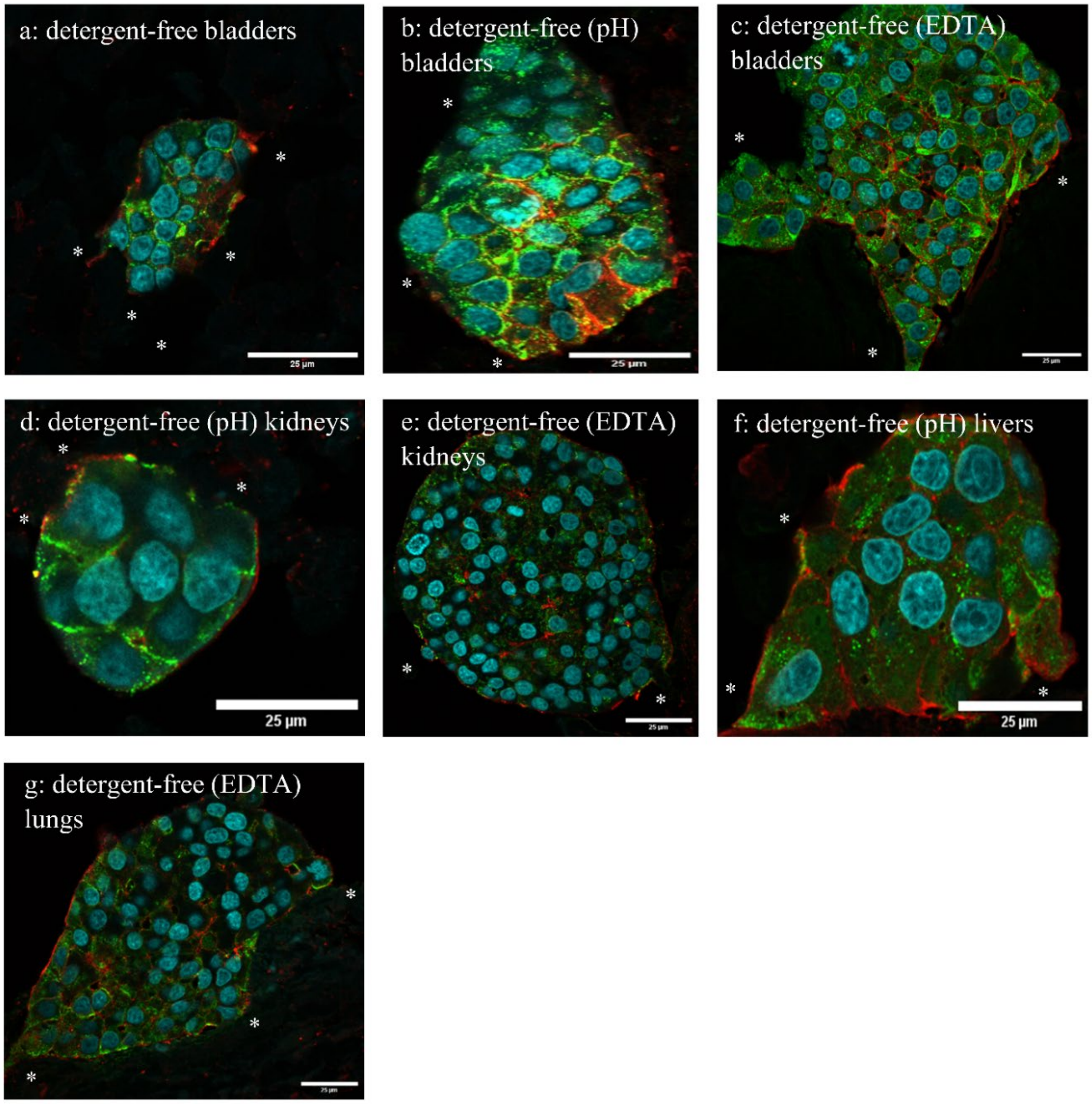

Supplementary Figure 1: Immunostaining for insulin (green) and actin (red) of INS-1 cells cultivated on the different organs. INS-1 cells on bladders ECM obtained with the a) bare detergent-free method, b) detergent-free method with pH treatment, and c) detergent-free method with EDTA treatment. INS-1 cells on kidneys ECM resulting from d) detergent-free (pH) and e) detergent-free (EDTA) treatments. INS-1 cells on livers ECM produced with the f) detergent-free (pH) method. INS-1 cells on lungs ECM obtained from the g) detergent-free (EDTA) treatment. White * indicates focal adhesion points of cells to the ECM. Scale bars indicate 25 µm. Representative images are shown in the figure; data were validated on three different porcine donors (N=3).
